# Process evaluation of implementation fidelity in a Danish health-promoting school intervention

**DOI:** 10.1186/s12889-018-6289-5

**Published:** 2018-12-27

**Authors:** Ane Høstgaard Bonde, Nanna Wurr Stjernqvist, Marianne S. Sabinsky, Helle Terkildsen Maindal

**Affiliations:** 1 Steno Diabetes Center Copenhagen, Health Promotion, Niels Steensensvej 6, 2820 Gentofte, Denmark; 2 Division of Nutrition and Risk Assessment, National Food Institute, Technical University of Denmark, Kemitorvet 2, 2800 Kgs. Lyngby, Denmark; 3 Division for Diet, Disease Prevention and Toxicology, National Food Institute, Technical University of Denmark, Kemitorvet 2, 2800 Kgs. Lyngby, Denmark; 4 Department of Public Health, Aarhus University, Bartholins Allé 1, 8000 Aarhus C, Denmark

**Keywords:** Health promotion, School, Complex intervention, Implementation, Process evaluation

## Abstract

**Background:**

“We Act” is a health-promoting school intervention comprising an educational, a parental and a school component. The intervention was implemented in 4 Danish public schools with 4 control schools. The objectives were to improve pupils’ dietary habits, physical activity, well-being and social capital using the Investigation, Vision, Action & Change (IVAC) health educational methodology. The target group was pupils in grades 5–6. The purpose of this study was to evaluate implementation fidelity and interacting context factors in the intervention schools.

**Methods:**

The Medical Research Council’s new guidance for process evaluation was used as a framework. Data were collected concurrently and evenly at the 4 intervention schools through field visits (*n* = 43 days), questionnaires (*n* = 17 teachers, 52 parents), and interviews (*n* = 9 teachers, 4 principals, 52 pupils). The data were analysed separately and via triangulation.

**Results:**

A total of 289 pupils participated, and 22 teachers delivered the educational component in 12 classes. In all schools, the implementation fidelity to the educational methodology was high for the Investigation and Vision phases as the teachers delivered the proposed lessons and activities. However, the implementation fidelity to the Action & Change phase was low, and little change occurred in the schools. The pupils’ presentation of their visions did not work as intended as an impact mechanism to prompt actions. The implementation of the parental and the school components was weak. The main context factors influencing implementation fidelity were a poor fit into the school-year plan and weak management support.

**Conclusions:**

Although ‘We Act’ was designed to comply with evidence- and theory-based requirements, IVAC and the health-promoting school approach did not result in change. The time dedicated to schools’ preparation and competence development may have been too low. This must be considered in future process evaluation research on health-promoting schools and by school health promotion administrators when planning future school interventions.

**Trial registration:**

ISRCTN85203017

**Electronic supplementary material:**

The online version of this article (10.1186/s12889-018-6289-5) contains supplementary material, which is available to authorized users.

## Background

Childhood represents an important life stage for the development of healthy nutritional and physical activity behaviour, and evidence indicates that health behaviour continues from childhood into adulthood [1, 2]. The health-promoting school approach is a comprehensive answer to the challenge of promoting children’s health in the school setting [3]. To be effective, health-promoting school interventions must have multiple components and address the following three areas: health education in the curriculum, changes to the school environment and parental and/or community engagement. However, a systematic Cochrane review assessed the quality of the evidence for the effect as low to moderate [4].

The democratic health education approach has developed within the context of the health-promoting school in Europe [5, 6]. The Investigation, Vision, Action, Change (IVAC) educational methodology was developed as a framework for working with pupils’ participation, towards action competence in health by actively involving pupils in change processes in the school or community [7]. Research on the IVAC methodology, including a multiple case study with five schools in five different European countries [8] and a cluster-randomized study in 16 schools from one European city [9], has demonstrated positive results on children’s action competence and body mass index, respectively. However, evidence on the implementation and effects of this educational methodology is still scarce.

On this basis, the “We Act - together for health” study (hereafter, “We Act”) was designed with the purpose of developing, implementing and evaluating a health-promoting school intervention. We Act was a quasi-experimental study with four intervention schools matched with four control schools. The target group was pupils in grades 5–6 (ages 10–13 in Danish schools). The We Act intervention was designed as a curriculum integrated intervention linking to the school’s core business of education as recommended [10, 11], thus avoiding school health promotion to be viewed by schools and teacher as yet another “add-on” in a busy school life [12]. The intervention objectives were to improve pupils’ diet, physical activity, well-being and social capital through developing their action competence and promoting a healthy school environment. We Act was a complex intervention, as it comprised multiple interacting components to be implemented at different levels [13].

Research has shown that complex and multi-component interventions are seldom implemented as planned [14]. This is particularly true in school settings, where adaptions may be inevitable and even beneficial [15]. However, a study on teachers’ implementation of a prevention program found that just under half of the adaptions teachers made were positive, as consistent with the program’s objectives, while the rest were detracting and assessed as negative [16]. Therefore, process evaluations are necessary to explain and understand intervention results. Evaluating the process helps prevent type III error, which refers to drawing a wrong conclusion about the effects of an intervention [17]. Interventions may have limited effects because of design weaknesses, implementation shortcomings or both [18]. Common features in process evaluations of intervention-implementation are reach, dose, adherence, adaptions, fidelity, quality of delivery, participant responsiveness, and context [13, 14, 17, 18]. There remains no consensus on how best to define and divide these subcomponents [19]; however, “fidelity” is generally understood as the extent to which an intervention has been implemented (i.e., delivered) by the providers as planned and as intended by its developers. The Medical Research Council’s recent guidance for process evaluation of complex interventions summarizes the state of the art on the issue: “Capturing what is delivered in practice, with close reference to the theory of the intervention, can enable evaluators to distinguish between adaptions to make the intervention fit different contexts and adaptions that undermine the intervention fidelity” [19].

Therefore, the purpose of this study is to evaluate the implementation fidelity to the proposed We Act intervention by examining how the components were delivered in practice and to identify the interacting context factors.

### The We Act intervention

**Fig. 1:**
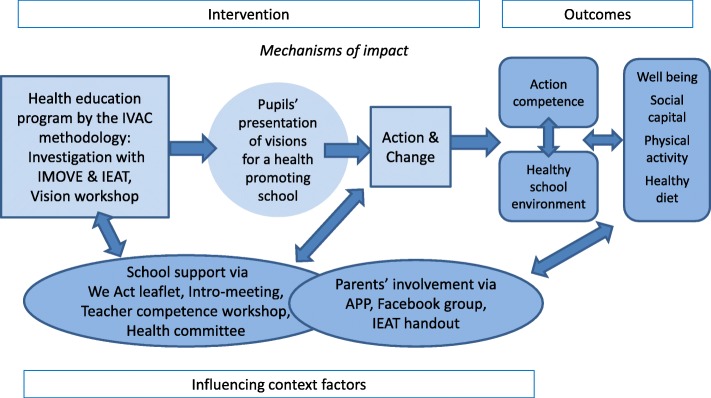
Intervention theory of ‘We Act – together for health’

The process evaluation focuses on the implementation of We Act in the four intervention schools from November 2015 to June 2016. The intervention theory of We Act was that pupils participating in health education following the IVAC methodology will develop visions for a health-promoting school and present them to a broad school audience. This is a key impact mechanism that intends to trigger pupils’ involvement in actions and changes towards a healthy school environment, which will lead to pupils’ action competence, a healthy diet, physical activity, social capital and well-being in (Fig. 1). The intervention consisted of three components as the three pillars of the health-promoting school [4].

The school component comprised the following elements: a) a We Act leaflet informing the school community about the intervention, b) introductory meetings with principals and key teachers to prepare the conditions and agree upon the implementation process, c) competence workshops for all involved teachers, principals and other resource persons at each school, and d) the formation of a health committee with the primary task of supporting the transition from pupils’ visions to actions at the classroom and school levels.

The educational component comprised four health education programmes called ‘IMOVE’,’ IEAT’, ‘Vision’ and ‘Action & Change’. They are integrated into the curriculum and lessons of maths and Danish language and developed to fulfil national educational objectives and health education objectives for grades 5–6. The programmes follow IVAC. This includes, first, the Investigation phase, where pupils investigate their physical activity with step counters (IMOVE) and their food intake with a log book (IEAT), followed by learning and critical dialogue in the next week. Second is the Vision phase, where pupils elaborate their visions for a health-promoting school and present them to a school audience; this represents the transition to the last phase, Action & Change, where pupils work to realize their visions with the support of teachers, health committee school management and parents.

The parental component comprised the following: a) an app called Healthy Kids Denmark©, which provides inspiration for packed lunches, b) a homepage and a Facebook group for communication among parents, and c) a handout, called “My food and meals in school”, to be created by the pupils during the IEAT lessons and taken home and discussed with their parents.

The material to guide the process included teacher guides, pupil assignments, step data sheets, food log books and a vision poster template. The IVAC educational process was proposed to include 40 lessons over 2–4 months. The We Act intervention and material were developed by a team of health promotion researchers in active cooperation with schools, teacher and pupils. The IMOVE program was developed in 2013–14 [20, 21]. IEAT, the Vision workshop and We Act as an IVAC process and with the 3 components were developed and pilot tested in 2015 [22].

## Methods

### Process evaluation

We used the Medical Research Council’s new guidance for process evaluation of complex interventions as a framework [19]. The key evaluation features regarding implementation fidelity were Reach, Dose, Delivery and Context (Table 1). Implementation fidelity was understood as reaching the target groups with the proposed dose of lessons and activities and the intended pupil participation according to the IVAC methodology. Context included anything external to the intervention that may directly or indirectly affect the implementation or its outcomes [17, 19]. Among the different levels of context factors influencing implementation quality in schools, we focused on school-level factors, leaving out macro-level and individual-level [23].

**Table 1:** Key features in the process evaluation of ‘We Act – together for health’

Evaluation feature	Evaluation question	Indicator of fidelity	Data source
Reach	Were the target groups of pupils, teachers, principals and parents reached as proposed?	- Proposed pupils and grades were involved in We Act	Field visits (=43) Questionnaires to parents (*n* = 52)
		- Teachers assigned and delivered as proposed	
		- Parents informed; used app, Facebook, and webpage; received IEAT handout; participated in pupils’ presentation of visions	
		- Principals participated in introduction meetings, competence workshop and pupils’ presentation of visions	
		- Health committee formed and supported the action phase.	
Dose and delivery	Was the dose of lessons and activities delivered as proposed, and with the intended pupil participation according to the IVAC methodology?	Investigation with IMOVE: 6 lessons over 2 weeks	Field visits (=43) Questionnaires to parents (*n* = 52) Interviews with teachers (*n* = 9) and principals (*n* = 4 principals in 7 interviews) Focus group interviews (=12) with pupils (*n* = 52)
		Investigation with IEAT: 9 lessons over 2 weeks (same 2 weeks as IMOVE)	
		Vision Workshop: 12 lessons over 2 weeks (immediately after IMOVE/IEAT)	
		Action & Change: 12 lessons over 1–3 months	
		- It worked well in practice that pupils collected their own data on steps and food intake	
		- All assignments were used for IMOVE and IEAT	
		- Pupils elected IMOVE managers (indicator of participation)	
		- Pupils worked with the IEAT handout (indicator of parent involvement)	
		- Steps for the Vision Workshop were followed and pupils participated actively	
		- Pupils presented their visions to an audience outside the class	
		- Pupils participated in actions	
Context	What context factors interacted and influenced the implementation fidelity?		All above
	How consistent were the adaptions with the intervention theory?		

### Data collection

Data were collected concurrently and evenly at the four schools during the implementation period by field visits, classroom observations, questionnaires and interviews according to the principles for process evaluation and mixed methods research [17, 24].

We conducted four introductory meetings and participated in four competence workshops facilitated by an external health-promoting school consultant. We thereby built a sense of rapport with the school actors [25]. During the implementation period, we visited each school several times and collected data through field visits, questionnaires and interviews. We addressed all key participants as informants to capture data on the comprehensive intervention.

The purpose of the field visits (*n* = 43) was to collect data on the implementation process in each school and to experience the interaction between teachers, pupils, and the We Act material. Capturing the influence of the context is one of the strengths of the observation method [26]. Our role in the field was as participating observers. For each visit, we took notes in a log book and an observation diary.

We created a short questionnaire for teachers regarding their implementation of each of the educational programmes, IMOVE, IEAT, Vision Workshop, and Action & Change. The teachers received the corresponding questionnaire the week after completing each programme. Twenty teachers received 31 questionnaires, as some of the teachers delivered two programmes. Seventeen questionnaires (55%) were returned from 14 teachers (70%). The questionnaire regarding Action & Change was never distributed, as none of the teachers completed this phase within the period of implementation.

By the end of the implementation period, the parents of all pupils (*n* = 302) were given an online questionnaire (returned by 52, response rate 17%) about their knowledge and use of the parental support elements.

We carried out focus group interviews with pupils from all classes in two of the schools (12 groups, 52 pupils) to learn their perspectives on their participation. In this paper, we report pupils’ assessment of their engagement in each educational programme, as indicated by a happy, medium or sad face.

We invited the four principals and the 12 teachers delivering the Vision Workshops (who had also delivered either IEAT or IMOVE) to a personal interview by the end of the implementation period (3 principals and 9 teachers from all four schools were interviewed). The purpose was to explore implementation fidelity and quality of delivery by asking how they used the We Act material and unfolded the IVAC methodology and pupils’ participation in practice. In October of the next school term, the four principals participated in a follow-up interview concerning the status of the actions derived from the pupils’ visions (see Additional file 1: Table S1).

### Data analysis

The data analysis occurred in several steps and followed the principles and procedures for analysis of qualitative data [24, 25]. First, we performed a systematic reading of the field notes and the compiled questionnaires given to teachers and parents for an overview of the implementation process of each component in the four schools. This provided a preliminary answer to the evaluation questions regarding reach, dose, fidelity and context influence. Next, the transcribed interviews with teachers and principals were read and coded in multiple rounds. We coded for units related to the elements of the IVAC methodology and for which part of the We Act components the particular unit belonged to. This provided content regarding the process of delivery, the reasons for deviation from the proposed context, and the interaction with the context. Thereby the picture of implementation fidelity was expanded. The last step was a cross-cutting analysis of the comprehensive and systematized data material. Here, we assessed the identified adaptions to context as either consistent with the intervention theory or detrimental, and we drew specific findings and broader conclusions.

During the analysis process, we used the data sources to supplement one another. For instance, when a teacher had not returned a questionnaire, we looked in our field notes for information on when he had worked with the different parts of We Act, and we complemented questionnaire data with interview information about the same topic. We thereby triangulated our data by combining and comparing the information and results from different methods to provide a more comprehensive picture than either method could do alone [26].

### Ethics

The We Act study adheres to all Danish ethical standards of participant information, consent, confidentiality and data handling. It was approved by the Danish Data Protection Agency on April 18, 2015 (ref: 2015–41-420) and reported to the regional ethics committee for the Capital Region of Denmark Protocol no. H-7-2015-FSP1.

## Results

We present the findings as a summary across data and according to the evaluation features of reach, dose, delivery, context and implementation fidelity (Table 1).

### Reach

The target group of pupils in grades 5–6 was reached, and 3 classes from each intervention school participated for a total of 289 pupils in 12 classes (Additional file 1: Table S2). The principals assigned the proposed teachers to deliver the educational programme: 10 Maths teachers delivered IMOVE, 11 Danish language teachers led IEAT, the Vision Workshop, and Action & Change, and in one class, a support teacher was assigned instead of a Danish teacher. The principal or vice principal and the teachers participated as proposed in the four introduction meetings and four competence workshops, with a few exceptions. Three schools followed the proposal of inviting other resource persons to the competence workshop; three school nurses attended, but none of them participated any further.

All parents of participating pupils were targeted with the digital support elements, and according to the parental questionnaire (which had a poor response rate), the majority read the parent information letter. However, only few of them used the app and visited the homepage, and none joined the Facebook group. A third of the parents read the IEAT handout from their child. The field observation data showed that parents were invited to and attended the pupils’ presentation in 4 of the 12 classes.

### Dose and delivery

#### Investigation

The teachers delivered the IMOVE and IEAT programmes as proposed in the 12 classes, with some minor adaption (Additional file 1: Table S3): They performed a few more lessons than proposed and did not use all the assignments, with larger teacher variation in IEAT than in IMOVE. Most teachers found that it worked well in practice that pupils collected their own data to investigate physical activity and food intake and that the curriculum integration of health education into Danish and maths lessons fulfilled the double set of objectives as intended. For example, when pupils analysed their step data with the IMOVE material, they learned statistics and gained insight into their activity patterns, and when they analysed the “Pippi Longstocking goes to a coffee party” text from the IEAT material, they discovered and reflected on different rules and norms for meals in the class and in their families. The IEAT assignment about the official dietary guidelines, where pupils worked in groups, was underlined as participatory and engaging. Only two teachers made pupils elect IMOVE managers, which was a key participation element in IMOVE. Most teachers made pupils work with the IEAT handout, which was a key element of parent involvement. They found it a brilliant idea for pupils to make a handout explaining to their parents what they like in a packed lunch. However, the teachers stated that only few pupils took the handout home, and they did not follow up on it as proposed.

#### Vision

The teachers delivered the Vision Workshop in a few more lessons than the proposed 12, and all classes developed visions for a health-promoting school. Most of the steps suggested in the teacher’s guide were followed, starting with brainstorming and then voting democratically about which visions to continue with. Then, groups were formed, and the groups worked to concretize their visions with the support of the teachers. Finally, the groups prepared their presentations using the vision poster template, PowerPoint presentations and other creative presentation options.

All teachers found that the Vision Workshop engaged and motivated the pupils. For example, the pupils had investigated the possibilities and prices for their ideas on the internet, placed phone calls and made comparisons. One group wrote a letter to the municipality requesting more time for physical activity during school hours, and another group created and administered a survey among their fellow pupils about bullying. The pupils themselves rated their engagement in the Vision Workshop with 90% happy faces (10% medium or sad faces); in comparison, they rated their engagement in IMOVE and IEAT with 48 and 25% happy faces, respectively (Additional file 1: Table S4).

The pupils developed a total of 61 visions for a health-promoting school. Most of the visions were about improving food supply and opportunities for physical activity in school. The visions also described social aspects of togetherness, fun and having a good time while eating or playing; thus, they reflected a holistic concept of health (Additional file 1: Table S5). As the culmination of the Vision Workshop, the pupils presented their visions in all schools, and 10 of 12 classes presented their visions to an audience external to the class. In the audience were parents and/or a fellow class, and in 9 classes, the principal or vice principal also attended. However, it did not work as an impact mechanism to trigger the transition to the next phase, as the management had neither facilitated the formation of a health committee nor given others the task of supporting teachers and pupils in the Action & Change phase.

#### Action & Change

The proposal for the Action & Change phase was to perform 8–12 Danish lessons over 1–3 months and work at the class level or school level, depending on the nature of the pupils’ visions. One teacher delivered this phase in her class, as proposed, by facilitating a friendship eating event with a younger class. Further, a group of pupils from this class requested soap dispensers for the classroom to improve hygiene; this request coincided with a case of hepatitis in the school and made the management quickly provide the soap dispenser. In another school, two teachers started working with their pupils on actions in a few lessons but then stopped in order to prepare for a Danish test and other curricular activities. The rest of teachers did not work with actions at the class level, and in only one school did the pupils’ visions reach the school-board level.

At the time of the follow-up interviews, very little action had occurred in all four schools, and pupils had not been involved in the few environmental changes that were made, such as provision of dustbins, soap dispensers and a previously planned parkour lane (Additional file 1: Table S5).

### Context

In all schools, competition and workload from other educational activities were context factors that influenced the implementation fidelity negatively as it caused a delayed start of the We Act programs. In only one class did the teacher start implementing We Act directly following the competence workshop in January, as proposed, leaving time for all the IVAC phases (Additional file 1: Table S6). The other teachers started in February, March or April because of previously planned activities, such as a theatre performance or a natural science project. The latter took longer than planned and ended up competing with We Act for teachers’ and pupils’ time and attention. In one school, pupils presented their visions just before the end of the school year; this left no time for the Action & Change phase and thus undermined the intervention, as any decision on action was postponed to the next school year. In one school, the three teachers chose to work together and fit the Vision Workshop into the schools’ yearly cross-disciplinary week, where they mixed pupils across the three classes. This adaption to context was per se consistent with the intervention theory, and the pupils were very engaged in the Vision Workshop; however, it undermined the proposal for the Action & Change phase, which was to work with pupils’ actions at the class level.

Teacher illness was another influencing context factor in all schools, but the schools coped with it in different ways. In one school, a Danish teacher of one of the We Act classes had a long-term illness. Thus, a support teacher was assigned and used support lessons instead of Danish lessons. The support teacher delivered IEAT and the Vision Workshop as any other teacher. Thus, apart from not being integrated into the Danish curriculum, this was an example of a positive adaption to an intervening context factor. In another school, two Danish teachers went on sick/ maternity leave after having delivered IEAT and two maths teachers who had delivered IMOVE took over and implemented the Vision Workshop. This also represented positive adaption. In a third school, the Danish teacher fell ill halfway through the Vision Workshop. A substitute teacher was assigned, but he did not finish the workshop; thus, the intervention was undermined, as the pupils were disconnected from the process.

The school year plan was an overriding context factor that influenced implementation fidelity. We Act was adapted to the year plan; however, the delayed start was not fully compatible with the intervention theory, as less or no time was left for the Action & Change phase. By the end of the process, teachers and principals reflected on the intervention’s comprehensiveness and concluded that We Act should have been introduced earlier to better fit with the school year plan.

Management support was an overriding context factor with a strong influence on the transition from visions to actions. Elements of management support were built into the school component by stressing principals’ presence at key events. However, this did not prompt the formation of a health committee or the support to bring pupils’ visions to action except in one school, where the visions reached the school board. The interacting context factor identified was that the vice principal invited the president of the school board to the pupils’ presentations. Subsequently, the two of them agreed to include the pupils and their visions on the agenda of a meeting with the entire school board, which took place a month later. In another school, the vice principal gave the list of pupils’ visions to the principal and expected him to bring it to the school board, but he took it only as a piece of information, and nothing occurred. In the third school, the vice principal had expected to revitalize the former existing health committee, but it just did not happen. She reflected that management should have assigned more resources to involve the whole school in We Act or, alternatively, assigned decision competence to the teachers and made clear to them that action and change should be kept within the existing budget. This would have been possible for some of the pupils’ visions, such as daily time for physical activity. The last school changed principals over the summer, and the new principal did not know details about We Act.

### Implementation fidelity and impact mechanisms

Taking a cross-cutting look at implementation fidelity in terms of reaching the target groups of the three We Act components, we found that implementation fidelity was quite high in the educational component, including the Investigation and Vision phases. The teachers delivered the proposed dose of lessons and activities with the intended pupil participation, with the exception of some minor omissions or adaptions that did not undermine the intervention. Thus, here, the curriculum integration and the interaction between the intervention and the classroom context worked well (Additional file 1: Table S7). The implementation fidelity regarding the Action & Change phase was low; only one teacher delivered this phase, even though the pupils in 10 classes presented their visions for a health-promoting school to an external school audience. Hence, the intended impact mechanism to prompt the transition from Vision to Action & Change did not work. The first elements of the school component, the introductory meeting and competence workshop, were implemented as proposed. However, the health committees were not formed, and management support for the Action & Change phase was low. Thus, the outcomes of pupils’ action competence and a healthy school environment were not prompted as intended.

## Discussion

This process evaluation of We Act adds to the research by examining the challenges of implementing a complex, three-component health-promoting school intervention that uses the IVAC methodology. We found that in all schools, implementation fidelity to the educational component was high in the Investigation and Vision phases but low in the Action & Change phase. The pupils presented their visions in all schools, but the presentations did not work as an impact mechanism to prompt pupils’ actions and school environmental change as intended. The parental component was weakly implemented. Implementation fidelity to the school component was low regarding management support, except in one school, and very little change occurred in all schools. This is in contrast to findings from a health-promoting school project in Cyprus with the IVAC methodology in which action and environmental change was achieved in 6 of 7 participating schools [27].

We Act was designed according to the health-promoting school and democratic health education approach and included most of the evidence-based requirements for context to make the health-promoting school approach work, as described by Rowling and Samdal [28]: preparing for school intervention, professional development, leadership, pupil participation, partnership with parents, and policy anchoring. Below, we discuss these six context factors in relation to our findings and similar studies on the topic.

***Preparing*** for a health promotion intervention includes establishing a leadership commitment, planning how to incorporate the intervention into the school’s annual plan and forming a coordination committee. All of these factors were considered in the We Act leaflet and introduction meeting; however, they may have not been considered with the sufficient “dose”, emphasis or time allocated, as low management support and difficulty in fitting We Act into the school year were among the negatively influencing context factors. Hence, this study draws attention to the importance of preparing the context – also called “creating ownership” – as a condition for a health-promoting school to flourish [29].

***Professional development*** was included in We Act as a three-hour competence workshop in each school, and the attendance and appreciation among teachers and school management were high. Other school intervention studies provided longer teacher training, such as a two-day seminar in a Danish outdoor education intervention [30] or an initial training followed by continuous support to teachers via six meetings over a two-year intervention period in Spanish schools [9]. From this perspective, the We Act “dose” may have been too low, especially considering that the notion of “Action & Change” has been found to be very challenging for teachers, as it is not embedded in educational practise and school context [8].

***Leadership***, a crucial factor for the success of a health-promoting school, was considered in We Act by encouraging principals to take a leadership position and establish a committee for coordination and support. No such committee was present in any of the schools. The We Act intervention design did not include a support system, as recommended by Domitrovich et al. [23] and practised in a Dutch programme that nominated local school coordinators who were supervised every 2 months by a school health promotion advisor [31]. Insufficient support may explain the low implementation fidelity in We Act.

***Pupil participation*** was intended to be a core mechanism in We Act; however, with exception of the Vision Workshop, pupils’ participation may not have been “genuine participation” as described in the Democratic Health Education approach. For participation to be genuine, pupils must be involved in decision making about the process and contents of health learning, and teachers must be open to pupils’ ideas about changes in the school environment and facilitate a change process where pupils, with support from teachers, get action experience [6]. The teachers and the school management may not have been sufficiently prepared for this in We Act.

***Partnership with parents***, which is crucial for an intervention that includes a focus on children’s lunch brought from home, was included as a separate component with several elements. However, parent involvement was low, which has been proven by both this study and others to be one of the most challenging areas of implementing the health-promoting school approach [12, 32].

***Policy anchoring*** was not a requirement for schools to sign up for the intervention, but the We Act leaflet mentioned a health policy as a possible outcome of the process. One school had a health policy, but it did not seem to influence the implementation. This is in contrast to Swedish school managers who consider the legal duty and a school-health policy to be important for their implementation of health promotion [33]. We did not look into national policy anchoring, as we left out macro-level influencing context factors. Including this could have contributed to an underlying societal explanation of the weak implementation of We Act at the school level, as readiness for health promotion requires the education sector to take responsibility for health promotion in schools [3]. This is not the case in Denmark, according to Simovska et al. [34], who note a gap between the national school health policy goals and local educational practice.

Our study is novel as it is one of the first to apply the Medical Research Council’s new comprehensive framework [19] for a process evaluation of a complex health promotion intervention in a school setting. The comprehensiveness of our process evaluation is a strength but also a limitation, as it is not possible to perform an in-depth analysis of all the data collected regarding all features and all informant groups. Another option would have been to cut the process evaluation results into slices for each component and informant, such as presenting the evaluation of the curriculum component via teacher interviews in one paper [35] and the parental component via parent surveys in another paper [36]. Our choice provides an overview of the complexity, but the presentation of each aspect is brief.

## Conclusions

Although We Act was designed to comply with evidence- and theory-based requirements, IVAC and the health-promoting school approach did not institute change in the local school context. In particular the action phase, was not implemented as proposed. This knowledge is now available for understanding the effect of We Act, a quasi-experimental study, on pupils’ diet, physical activity, and well-being; and the negative effect found on school belonging as an indicator of social capital [37].

The “dose” of time for schools to prepare for the intervention and to develop staff competence in health promotion with the IVAC methodology may have been too low. Based on this study, which focused on the implementation process, we suggest that future process evaluation research on school health promotion addresses the early stages of communication and planning with schools on their participation in health promotion, to explore what preparation and how much is “enough”. The research team should include participants from both health and education sector. Future research may also consider strategies for involving parents and explore why this is so difficult that studies, including our, consistently describes this as the most challenging and least successful intervention component. Finally, public health and school administrators should consider, when planning future health-promoting school interventions, to allocate more time and resources for the initial planning and commitment phase, involving representatives from health and education sectors as well as parents.

## Additional file

Additional file 1: **Table S1.** Summary of collected data. **Table S2.** Participating pupils by school and grade. **Table S3.** Lessons proposed vs lessons delivered. **Table S4.** Pupils’ rating with smileys of their engagement. **Table S5.** Visions and actions by school. **Table S6.** Implementation period of We Act by school and class. **Table S7.** Implementation fidelity and adaptions. (DOCX 56 kb)

## Abbreviations

IVAC: Investigation Vision Action & Change
