# Supplementary material for: Process evaluation of implementation fidelity in a Danish health-promoting school intervention
Source: BMC Public Health. 2018 Dec 27;18:1407. doi: 10.1186/s12889-018-6289-5 (PMC6307196; doi:10.1186/s12889-018-6289-5)
Supplement: Supplementary file 1 — Table S1. Summary of collected data. Table S2. Participating pupils by school and grade. Table S3. Lessons proposed vs lessons delivered. Table S4. Pupils’ rating with smileys of their engagement. Table S5. Visions and actions by school. Table S6. Implementation period of We Act by school and class. Table S7. Implementation fidelity and adaptions. (DOCX 56 kb) [file 12889_2018_6289_MOESM1_ESM.docx]

**Table S1. Summary of collected data**

| \| **Method and informant** \|  \| **Jan-May 16** \| **June 16** \| **Oct 16** \| \| --- \| --- \| --- \| --- \| --- \| \| **Field visits** \| *No of meetings/observation visits*: \|  \|  \|  \| \|  \| Meetings with teachers and/or principals \| 10 \|  \|  \| \|  \| Classroom observation \| 26 \|  \|  \| \|  \| Vision presentation observation \| 7 \|  \|  \| \| **Questionnaires** \| **Teachers** \|  \|  \|  \| \|  \| Invited \| 31 \|  \|  \| \|  \| Responded \| 17 \|  \|  \| \| **Questionnaires** \| **Parents** \|  \|  \|  \| \|  \| Invited \|  \| 303 \|  \| \|  \| Responded \|  \| 52 \|  \| \| **Focus group interviews** \| **Pupils** \|  \|  \|  \| \|  \| Invited for 12 focus groups \|  \| 60 \|  \| \|  \| Participated in 12 focus groups \|  \| 52 \|  \| \| **Individual interviews** \| **Teachers** \|  \|  \|  \| \|  \| Invited \|  \| 12 \|  \| \|  \| Participated \|  \| 9 \|  \| \| **Individual interviews** \| **Principals/vice principals** \|  \|  \|  \| \|  \| Invited \|  \| 4 \| 4 \| \|  \| Participated \|  \| 3 \| 4 \|   **Table S2. Participating pupils by school and grade** |  |
| --- | --- | --- | --- | --- | --- | --- | --- | --- | --- | --- | --- | --- | --- | --- | --- | --- | --- | --- | --- | --- | --- | --- | --- | --- | --- | --- | --- | --- | --- | --- | --- | --- | --- | --- | --- | --- | --- | --- | --- | --- | --- | --- | --- | --- | --- | --- | --- | --- | --- | --- | --- | --- | --- | --- | --- | --- | --- | --- | --- | --- | --- | --- | --- | --- | --- | --- | --- | --- | --- | --- | --- | --- | --- | --- | --- | --- | --- | --- | --- | --- | --- | --- | --- | --- | --- | --- | --- | --- | --- | --- | --- | --- | --- | --- | --- | --- | --- | --- | --- | --- | --- |

| **School** | **No. Pupils Grade 5** | **No. Pupils Grade 6** | **Total no. Pupils** |
| --- | --- | --- | --- |
| A | 40 | 34 | 74 |
| B | 68 | 0 | 68 |
| C | 22 | 54 | 76 |
| D | 71 | 0 | 71 |
| Total no. Pupils | 201 | 88 | 289 |
| Each school participated with 3 classes. 9 classes were Grade 5. | | | |
| One from school A and two from school C were Grade 6. | | | |

**Table S3. Lessons proposed vs lessons delivered**

**Table S4. Pupils’ rating with smileys of their engagement**

| **Pupils' rating of their engagement in each program** | | | | | |  |  |  |  |  |  |  |  |
| --- | --- | --- | --- | --- | --- | --- | --- | --- | --- | --- | --- | --- | --- |
| Each of the 12 focus groups could give four smileys of any colour to each of the three programs. | | | | | | | | | |  |  |  |  |
|  |  |  | |  |  |  |  |  |  |  |  |  |  |
| Program |  | IEAT | |  |  |  | IMOVE |  |  |  | Vision Workshop |  |  |
| Smiley | Sad | | Medium | Happy |  | Sad | Medium | Happy |  | Sad | Medium | Happy |  |
| Score (af 4) | 0,9 | | 2,1 | 1,0 | 4,0 | 0,4 | 1,7 | 1,9 | 4,0 | 0,1 | 0,4 | 3,5 | 4,0 |
| Procent | 23% | | 52% | 25% | 100% | 10% | 42% | 48% | 100% | 2% | 9% | 89% | 100% |
|  |  |  | |  |  |  |  |  |  |  |  |  |  |
|  |  | IEAT | |  |  |  | IMOVE |  |  |  | Vision workshop | |  |
| \|  \| \| --- \| |  |  | |  |  |  | | | | \|  \| \| --- \| |  |  |  |
|  |  |  | |  |  |  |  |  |  |  |  |  |  |
|  |  |  | |  |  |  |  |  |  |  |  |  |  |
|  |  |  | |  |  |  |  |  |  |  |  |  |  |
|  |  |  | |  |  |  |  |  |  |  |  |  |  |
|  |  |  | |  |  |  |  |  |  |  |  |  |  |
|  |  |  | |  |  |  |  |  |  |  |  |  |  |
|  |  |  | |  |  |  |  |  |  |  |  |  |  |
|  |  |  | |  |  |  |  |  |  |  |  |  |  |
|  |  |  | |  |  |  |  |  |  |  |  |  |  |
|  |  |  | |  |  |  |  |  |  |  |  |  |  |
| The pupils themselves rated their engagement in the vision workshop with 90% happy smileys (10% sad or medium smileys) | | | | | | | | | | | | |  |
| compared to their engagement in IMOVE and IEAT which were rated with 48% and 25% happy smileys respectively. | | | | | | | | | | | |  |  |

**Table S5. Visions and actions by school**

| **School** | **Visions put into action** | **Visions with some progress** | **Visions not put into action** | **No. visions** |
| --- | --- | --- | --- | --- |
| School A  (2 classes) | Better sleep campaign  *(A group of girls hung up posters during the Vision Workshop)* | Skating slope  *(Left with the manager of the after-school center)* | Breakfast together  Fruit outlet/a canteen (x2 groups)  Morning exercise daily for 20 minutes  A tree for the class  Parkour lane  Artificial grass football lane  Home economics/cooking with a smaller class. | 10 |
| School B  (3 classes) | Better bathing facilities to the gym  *(New curtains and soap dispensers provided)* | Better outdoor areas  *(The school was already working on this and will take on some of the pupils’ visions)*  A canteen  *(The school and pupils’ council were already working to improve the lunch supply)*  Healthy snack outlet  *(The board president has started on this)* | Fruit tree and berry plantation  Cold drinking water dispensers  More physical activity and physical education  Cleaner school environment, more dustbins  Week where we teach a smaller class  Stopping bullying  Fridges and lockers | 6*  13 |
| School C  (2 classes) | Friendship meals with another class  *(One meal realized)*  Soap dispensers at sinks in our classroom  *(Provided)* |  | Daily morning exercise (x3)  Grass football lane/ improved lane (x3)  Fruit outlet, salad bar (x2)  Improved lunch outlet  Exercise wheel & power fitness lane  Cosy places to sit and eat outside | 14 |
| School D  (3 classes) | Fridge in the classroom for our packed lunch  *(Provided by a parent)*  More dustbins (*Provided)*  Parkour lane  *(Provided - already planned before We Act*) | Friday fruit in the class  *(The teacher brought this vision to the parents who are working on it).*  Better/lager canteen:  *The school and the pupils council was already working with this)* | More physical activity during school hours (x6)  Breakfast outlet  More equipment for the play ground  Cosy places to eat  Newsletter  More excursions out of school  Cleaner toilets | 18 |

*) Regarding school B: Six visions were democratically elected by the pupils (among 19) and presented for the principal and head of school board.

**Table S6. Implementation period of We Act by school and class**

| **Table S7. Implementation fidelity and adaptions** | |  |
| --- | --- | --- |
|  |  |  |
| **Components and subcomponents** | **Assessment of implementation fidelity and examples of deviations and adaptions** | **Label*** |
| **Educational component** |  |  |
| IMOVE | Implemented with minor deviation: Teachers delivered 1 lesson more than proposed | b |
|  | Not as participatory as intended, as most teachers omitted to involve pupils as IMOVE managers | b |
| IEAT | Implemented with minor deviation: Teachers delivered 3 lessons more than proposed, and not all assignments delivered | b |
|  | Not as parental involving as intended, as not all teachers worked with the handout to parents and not all pupils took it home. | b |
| Vision workshop | Generally implemented as proposed. A major deviation/adaption in school B was to mix the 3 classes in the vision workshop. | b/c |
|  | This is per se consistent with the vision workshop, but detrimental to the following action phase, proposed for class level. |  |
|  | A detrimental deviation was that 2 classes (school A and C respectively) did not present their visions for an external audience, | d |
|  | and was thus de-connected from the next phase. |  |
| Action & Change | Not implemented as proposed with pupils in any school. In school B, the visions were taken to the school board, and the pupils | d/e |
|  | were de-connected. In school D, they started until April, leaving no time for Action & Change before the end of the school year. |  |
| **School component** |  |  |
| Intro and compe- | Implemented as proposed in all schools and with the proposed participants, except minor deviation. A beneficial adaption in | a |
| tence workshop | school B and D: The principal assigned extra time for teachers' preparation of the We Act intervention |  |
| Management | In school B, the principal participated in all pupils’ presentations and the visions were brought to a higher management level. | a |
| support | In the other schools, the principals participated in some presentations and did not take action to further support the process. | d |
| Health committee | School A had a previous health committee with one member left. No meetings were held during the implementation period, | e |
| formation | nor was support provided to the process. In the other schools, no committee was formed or supported the process. |  |
|  |  |  |
| *) Label description | a) Implemented as proposed |  |
|  | b) Implemented with minor deviations, still consistent with the intervention theory |  |
|  | c) Implemented with major deviations as adaptions to context, still true to the intervention theory. |  |
|  | d) Implemented with detrimental deviations, diluting or undermining the intervention theory. |  |
|  | e) Not implemented at all, or to a very small degree. | **Cont.** |

| **Table S7. Continued** |  |  |
| --- | --- | --- |
|  |  |  |
| **Parental component** |  |  |
| App, Facebook group, homepage | Very little use of the support elements. | d |
| IEAT Handout | Minor deviation in school as not all teachers worked with the handout. Few parents received the handout and talked with their child about it. (b under educational component, d under parental component) | d |
| Lunch box | All pupils received it. Some parents were not aware about it. (Some pupils have two homes, some parents are not involved in preparing the packed lunch) | a |
| Participation in vision presentation | Parents assisted when invited. As in 2 classes in school A and 2 in school C. But they provided no further support. In the other classes, the parents were not invited. | a / e |
| **Core principles** |  |  |
| Curriculum-integration | IMOVE, IEAT and Vision Workshop: Generally integrated as proposed. An adaption in school B was to mix classes and use a cross-disciplinary week for the Vision workshop. An adaption in school C was to let a support teacher deliver instead of a Danish teacher, and use support lessons for We Act instead of Danish lessons. | a / c |
|  | Action & Change was not curriculum-integrated, and not implemented with pupils in any of the schools. | d |
|  | Timing in the school year: In 11 of 12 classes, We Act started later than proposes, leaving less time for Action & Change | e |
| Holistic health | IMOVE and IEAT: Focus is on physical activity and diet, however the material inspires to a holistic concept of moving/eating | b |
| concept | The vision workshop opens for any health related vision, and a holistic health concept is reflected in the pupils' visions | a |
|  | Action & Change: not implemented | e |
| Pupils' participation | IMOVE and IEAT: almost as proposed, however no "genuine" participation as most activities were adult-planned | b |
|  | Vision workshop implemented as proposed and pupils very active | a |
|  | Action & Change: not implemented | e |
| Action competence | In all schools, implementation towards action competence in pupils started well with high pupil engagement in the vision workshop, and pupils getting some action experience via participation in the vision workshop and presentation. But then pupils are de-connected, and the Action phase does not take place with pupils. (This principle overlaps with intervention outcome) | d |
| Parental involvement | Implementation fidelity was low as reach and involvement of parents was low (this overlaps with the parental component) | d |
| Healthy school environment | Implementation fidelity towards a healthy school environment was low. The We Act process did not contribute to a healthy(er) school environment, except very small changes like soap dispensers and dustbins. (This principle overlaps with intervention outcome) | e |
